# The neuro-immune axis in cancer: mechanisms of neural regulation of immunity within the tumor microenvironment to promote cancer progression

**DOI:** 10.3389/fimmu.2026.1838430

**Published:** 2026-09-01

**Authors:** Zhifan Ran, Chenghong Zeng, Jinli Han, Xinfeng Yu

**Affiliations:** 1 Department of Pharmacology, School of Basic Medical Sciences, Capital Medical University, Beijing, China; 2 The Sixth Clinical Medical School, Capital Medical University, Beijing, China

**Keywords:** cancer stem cells, immunotherapy, neuro-immune, neurotransmitters, perineural invasion, tumor innervation, tumor microenvironment

## Abstract

The tumor microenvironment (TME) is a complex and dynamic ecosystem composed of cancer cells, stromal cells, immune cells, and the extracellular matrix, which significantly influences the initiation, progression, and therapeutic response of cancer. Emerging evidence indicates a crucial interaction between the nervous and immune systems within the TME, forming a “neuro-immune axis” that profoundly impacts tumor biology. This review not only synthesizes current knowledge on this critical bidirectional crosstalk but also offers a fresh perspective by critically evaluating underappreciated aspects such as the neural differentiation of cancer stem cells and the challenges in translating preclinical findings into clinical therapies. On one hand, tumor cells actively promote nerve recruitment and remodeling through tumor innervation, neurogenesis, and perineural invasion. In turn, neural signals-including adrenergic, cholinergic, and sensory neuropeptides-orchestrate immunosuppression by modulating key immune cell populations such as T cells, macrophages, and myeloid-derived suppressor cells (MDSCs). Consequently, neural signaling promotes immune escape, tumor progression and therapeutic resistance. This review further critically evaluates the translational potential of targeting these neuro-immune interactions, highlighting combination strategies designed to enhance the efficacy of cancer immunotherapy.

## 1 Introduction

The tumor microenvironment (TME) is a complex and dynamic ecosystem that critically regulates tumor progression and therapeutic response. It is composed of malignant cells, diverse stromal and immune cells, and non-cellular components, all engaging in intricate crosstalk (1–3). While the roles of cancer-associated fibroblasts (CAFs), tumor-associated macrophages (TAMs), and metabolic reprogramming in fostering an immunosuppressive milieu are well-established (4–10), a pivotal but less explored component is the nervous system. Its integration into the TME, forming a neuro-immune axis, is now recognized as a major contributor to cancer pathogenesis and therapy resistance (11–14).

The nervous system actively regulates immune responses, and tumor-induced neurogenesis is linked to poor prognosis in various cancers (12, 15, 16). This neuro-immune crosstalk promotes an immunosuppressive environment, aiding tumor growth and metastasis (11, 17). Additionally, nerve-cancer interactions contribute to resistance to immune checkpoint inhibitors (18, 19). Understanding this axis opens new therapeutic avenues, offering a more integrated view of cancer as a neuro-immune-stromal entity (13, 20).

**Figure 1:**
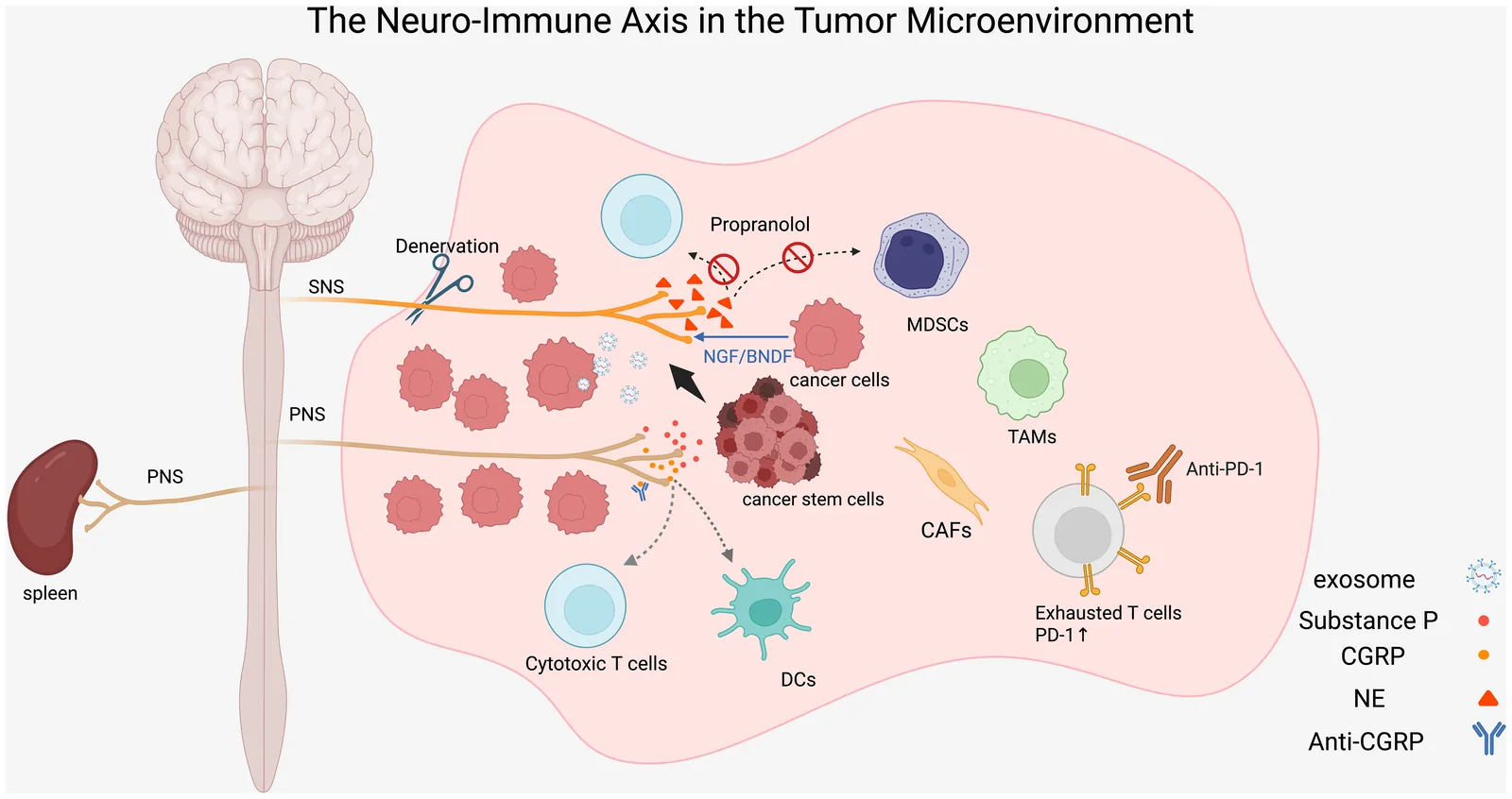
The neuro-immune axis in the tumor microenvironment. This schematic illustrates the complex interplay between the neural, immune, and cancer cell compartments within the TME. The TME is infiltrated by nerves and contains a heterogeneous population of cells, including cancer cells, CSCs, MDSCs, TAMs, CAFs, exhausted and cytotoxic T cells, and DCs. Both the SNS and parasympathetic nervous systems (PNS, originating from the brain, extend into the TME and connect with peripheral organs like the spleen. Nerves release neurotransmitters and neuropeptides, such as NE, CGRP and substance P, directly modulate immune cell function. Conversely, cancer cells secrete neurotrophic factors (e.g., NGF/BDNF) and exosomes to promote axonogenesis and nerve recruitment. Several therapeutic strategies targeting this axis are shown, such as pharmacological denervation, β-blockers (e.g., propranolol) to antagonize adrenergic signaling, and antibody-based agents (anti-CGRP, anti-PD-1) to block specific pathways.

This review explores the mechanisms of tumor innervation and neurogenesis. Cancer cells recruit and remodel nerves, and perineural invasion (PNI) is linked to increased disease aggressiveness (12, 21). Neurotransmitters and neuropeptides, such as adrenergic and cholinergic signals, influence immune cell functions, including T cells and macrophages (22, 23). Understanding these pathways can identify novel therapeutic targets to address immunotherapy resistance (19) (**Figure 1**).

## 2 Tumor innervation and neurogenesis in the TME

### 2.1 Cancer cells actively drive nerve recruitment and remodeling

Tumor innervation, or cancer-associated neurogenesis, is a process whereby cancer cells actively recruit neural progenitors and remodel neural structures to support tumor growth and progression (12, 21, 24, 25). This phenomenon is a critical hallmark of cancer, significantly contributing to tumor aggressiveness and poor patient outcomes (15, 26). Cancer cells secrete a variety of neurotrophic factors such as nerve growth factor (NGF), brain-derived neurotrophic factor (BDNF), and glial cell line-derived neurotrophic factor (GDNF), as well as axon guidance molecules (12, 15). These secretions promote nerve infiltration within TME, fostering a supportive neural network. The process, termed axonogenesis, is essential for establishing communication between the tumor and the nervous system, creating a neuro-immune oncology axis that influences tumor behavior (12, 16).

### 2.2 Cancer stem cells promote neural differentiation

In addition to recruiting existing nerves, recent research indicates that CSCs isolated from malignancies such as gastric and colorectal carcinoma can directly generate neurons within the tumor through the plasticity of CSCs. CSCs can differentiate into cells expressing neuronal markers and releasing neurotransmitters under specific conditions, as demonstrated in models of gastric and colorectal carcinoma (27). This capability suggests that CSCs may provide a localized, tumor-derived source of neural or neuroendocrine signals within the tumor mass, contributing to tumor neurogenesis. However, the physiological relevance and functional maturity of these CSC-derived cells *in vivo* require further investigation. It remains to be fully elucidated whether they integrate into functional neural circuits or primarily exert paracrine effects.​ Studies have shown that inhibiting this differentiation capacity suppresses tumor growth in mouse models, suggesting that targeting this process could be a novel therapeutic strategy (27). Thus, the neural transdifferentiation of CSCs represents a key mechanism by which tumors build an autonomous, self-reinforcing neural network to support their own growth and survival.

### 2.3 Tumor-derived exosomes mediate neural regulation

Exosomes, nanoscale extracellular vesicles released by tumor cells, also play a critical role in tumor innervation. Exosomes carry a cargo of proteins, lipids, and nucleic acids, including microRNAs (miRNAs), which can mediate intercellular communication (28, 29). Tumor exosomes carry neurotrophic factors and axon guidance molecules, delivering them to distant nerve fibers to promote axonogenesis and tumor innervation (29). Research has demonstrated that tumor-derived exosomes can induce neurite outgrowth in neuronal cell lines, such as PC12 cells, which is essential for tumor innervation in various cancer models. Moreover, Schwann cell-derived exosomes, such as those containing miRNA-21-5p, further increase tumor invasiveness and metastasis (30, 31). Blocking exosome release significantly reduces tumor innervation, indicating their critical role in the process (28).

### 2.4 PNI serves as a hallmark of aggressive tumors

PNI, the process by which cancer cells invade along nerves, is a well-established feature associated with tumor aggressiveness, poor prognosis, and increased metastasis (12, 15, 32). High intratumoral nerve density correlates with adverse clinical outcomes, including local recurrence and distant metastasis (12, 15, 32). This has been observed in multiple cancers, such as pancreatic ductal adenocarcinoma (PDAC), prostate cancer, and gastric cancer, where the degree of nerve infiltration serves as a prognostic biomarker (24, 32). Nerves provide a scaffold for cancer cells to migrate, facilitating tumor dissemination and metastasis. It is important to note that while this correlation is strong and reproducible, direct causal evidence that nerve ablation or pharmacological inhibition uniformly reverses poor prognosis in patients is still emerging, highlighting the need for further mechanistic and interventional studies. In addition, nerve-derived neurotransmitters and neuropeptides stimulate cancer cell proliferation, invasion, and epithelial-mesenchymal transition (EMT), contributing to an aggressive tumor phenotype (21, 33). For example, in PDAC, extensive PNI is associated with severe pain and poor prognosis, highlighting the dual role of nerves in both promoting metastasis and increasing patient morbidity (34). Thus, high nerve density in the TME is a critical factor driving malignant progression, positioning tumor innervation as a promising target for therapeutic intervention.

### 2.5 Cancer-induced nerve injury and immunotherapy resistance

**Figure 2:**
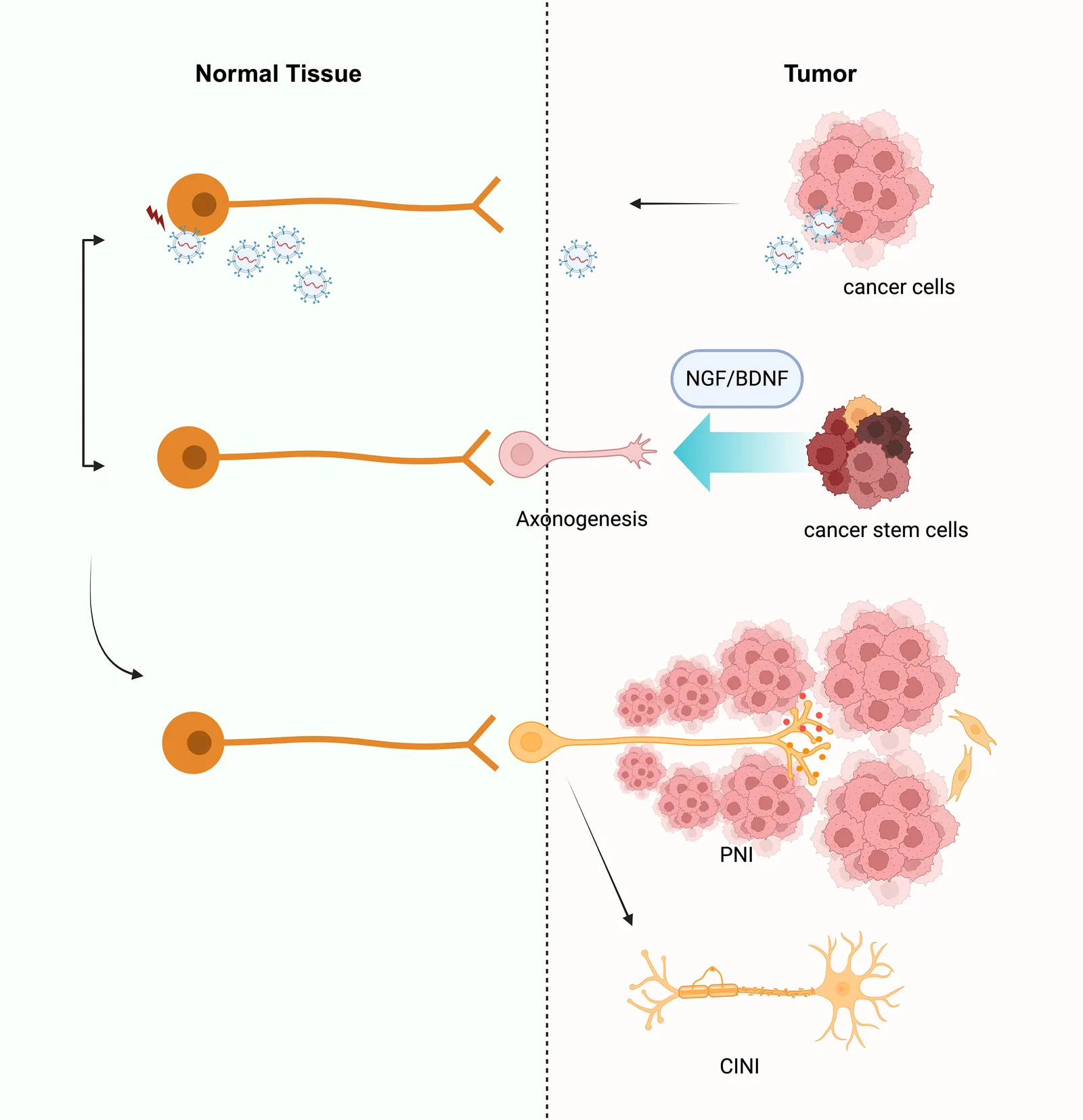
Mechanisms of tumor-induced innervation. This schematic illustrates the active process of tumor innervation. Within the tumor compartment, cancer cells secrete neurotrophic factors (e.g. NGF, BDNF) to induce axonogenesis. Tumor-derived exosomes systemically modulate neuronal states, facilitating nerve recruitment and growth. These mechanisms contribute to PNI, where cancer cell clusters co-opt and migrate along nerve fibers, using them as a scaffold for local spread. The figure also depicts CINI, characterized by the demyelination of nerve fibers. In contrast, the normal tissue shows a typical neuronal cell body with an intact, myelinated axon.

Cancer-induced nerve injury (CINI) also plays a crucial role in immunotherapy resistance. CINI, characterized by the degradation of nerve myelin, triggers chronic inflammation aimed at nerve healing. This inflammation contributes to an immunosuppressive environment through sympathetic-sensory neuronal plasticity and crosstalk within the TME, impairing the effectiveness of immune checkpoint inhibitors (ICIs) like anti-PD-1 therapy (18, 35). The sustained inflammation recruits immunosuppressive cells such as MDSCs and regulatory T cells (Tregs), which dampen anti-tumor immunity. Importantly, studies have shown that CINI-driven resistance to ICIs can be reversed through strategies like denervation or targeting specific molecular pathways (18), offering potential for new therapeutic combinations (**Figure 2**).

Together, the processes described above—tumor-driven nerve recruitment, CSC neural differentiation, exosome-mediated communication, and PNI—establish a rich neural network within the TME. The functional output of this network is largely mediated by the release of specific signaling molecules. The following section details how neurotransmitters and neuropeptides, released from these tumor-associated nerves, directly modulate the immune landscape to promote immunosuppression.

## 3 Neurotransmitters and neuropeptides modulate immune cell functions

The nervous system regulates immune responses within the TME primarily through the release of neurotransmitters and neuropeptides. These molecules function as key chemical messengers, modulating the activity of diverse immune cell populations (11, 14, 36). This intricate chemical communication represents cornerstone of the neuro-immune axis, directly impacting anti-tumor immunity and promoting immune evasion.

### 3.1 Adrenergic signaling: a potent suppressor of anti-tumor immunity

Adrenergic signaling, primarily mediated by norepinephrine (NE) and epinephrine released from sympathetic nerve endings, is a well-established pathway that profoundly suppresses anti-tumor immunity within the TME (37, 38).

#### 3.1.1 Sympathetic nerves release NE

Sympathetic nerve fibers extensively innervate the TME and release NE, which acts on adrenergic receptors (ARs) expressed on T cells, macrophages, dendritic cells (DCs), and MDSCs. This signaling modulates immune cell activity, trafficking, and cytokine production (36, 39). Activation of sympathetic nerves promotes tumor growth in multiple models, an effect that can be mitigated by inhibition of specific catecholaminergic neurons, indicating the direct involvement of NE in tumor progression (40). In cancer, NE predominantly contributes to an immunosuppressive microenvironment.

#### 3.1.2 Beta-adrenergic receptors promote immunosuppression

Beta-adrenergic receptor (β-AR) signaling, particularly through β_2_-AR, critically regulates MDSC frequency, survival, and immunosuppressive potential (39). Activation of β_2_-AR on MDSCs enhances their generation from precursor cells, promotes their survival, and upregulates immunosuppressive molecules, such as arginase-I and inducible nitric oxide synthase (iNOS), thereby directly inhibiting T cell function (39, 41). This effect is often mediated through the phosphorylation of STAT3, a key transcription factor involved in MDSC differentiation and function. This mechanism highlights a direct link between neural pathways and key immunosuppressive cell population within the TME.

#### 3.1.3 β-AR blockade enhances anti-tumor responses

Pharmacological blockade of β-ARs has emerged as a promising strategy to counteract adrenergic-mediated immunosuppression and improve therapy efficacy (42, 43). Propranolol, a non-selective β-blocker, has been shown to delay tumor progression and increase survival rates in various murine cancer models (43). Its anti-tumor effects are multifaceted, including reducing tumor angiogenesis and, critically, facilitating an anti-tumoral immune microenvironment characterized by increased T cell infiltration and reduced MDSCs (43). By attenuating the suppression of T cell priming in lymph nodes, β-AR blockade enhances CD8^+^ T cell activation and cancer vaccine efficacy (42). Furthermore, propranolol has been shown to upregulate PD-L1 on TAMs and enhance the efficacy of anti-CTLA4 therapy, indicating its potential as an adjuvant in immunotherapy (43).

### 3.2 Cholinergic signaling: a complex modulator of inflammation and immunity

Cholinergic signaling, mediated by acetylcholine (ACh) via nicotinic (nAChRs) and muscarinic (mAChRs) receptors, constitutes another major neuro-immune pathway. Largely associated with the parasympathetic nervous system, it plays a significant and context-dependent role in modulating inflammation and tumor immunity.

#### 3.2.1 Vagus nerve activation modulates immune cell activity

The vagus nerve, a key component of the parasympathetic nervous system, is a major regulator of systemic immune responses, particularly through its innervation of lymphoid organs such as the spleen (44, 45). Activation of the vagus nerve leads to the release of ACh in the spleen, which then modulates the activity of various immune cells. This “cholinergic anti-inflammatory pathway” can suppress pro-inflammatory cytokine production, such as TNF-α, and influence the overall immune milieu (45). While vagus nerve activation is often associated with anti-inflammatory effects (46), its precise role in tumor immunity is complex and context-dependent. For instance, during neuroinvasion in PDAC, elevated ACh levels suppress CCL5 activity via HDAC1, impairing CD8^+^ T cell recruitment. Simultaneously, this pathway directly interferes with interferon-γ (IFN-γ) synthesis and promotes Th2 over Th1 differentiation, thereby dampening cellular immunity (47).

#### 3.2.2 ACh/AChR signaling suppresses humoral immunity

ACh directly influences B cell function through specific AChRs, modulating antibody production and germinal center responses to suppress humoral immunity (48). Subtypes such as α7 and α9 nAChRs regulate TNF production and anti-inflammatory signaling, such as inhibition of ATP-induced IL-1β release, while α4 nAChR specifically inhibits CD19-B cell receptor colligation, which is critical for B cell survival and activation (47, 48). nAChRs are widely expressed in immune cells, with subunit composition varying by cell type and activation state, thereby critically shaping inflammatory and immune responses. For example, activated CD4^+^ T cells upregulate α4, α7, and β4 subunits, while CD8^+^ T cells increase α2, α4, and β4 subunits. Research also suggests that activation of α7 nAChRs on T cells leads to the upregulation of Tregs (47). Suppressing germinal center responses might reduce the generation of protective anti-tumor antibodies, potentially contributing to immune evasion.

### 3.3 Sensory neuropeptides and immunomodulation

Sensory neuropeptides, released by neuronal terminals within the TME, are potent regulators of local immunity. By binding to specific receptors on immune cells, these signaling molecules directly modulate immune cell recruitment, polarization, and functional activity, thereby exerting a profound influence on the anti-tumor immune response.

#### 3.3.1 CGRP

Nociceptor neurons infiltrating tumors release neuropeptides such as calcitonin gene-related peptide (CGRP), which significantly promotes immune evasion (22, 49). Specifically, CGRP induces exhaustion in cytotoxic CD8^+^ T cells via its receptor RAMP1, correlating with poorer prognosis (22). In medullary thyroid cancer, CGRP is highly expressed and linked to abnormal dendritic cell development and impaired T cell activity, further highlighting its immunosuppressive role (23). Genetic or pharmacological ablation of TRPV1-lineage neurons (a subset of nociceptors), silencing of nociceptors, or antagonism of the RAMP1, have been shown to reduce tumor growth and increase survival in mouse models, suggesting the direct immunosuppressive role of nociceptor-derived CGRP (22).

#### 3.3.2 Substance P

Substance P (SP), another neuropeptide often co-released with CGRP from sensory neurons, is known to modulate inflammatory responses and can affect the function of macrophages, T cells, and DCs (49). SP typically acts via the neurokinin-1 receptor (NK1R) and can promote pro-inflammatory responses, angiogenesis, and tumor cell proliferation, depending on the context.

#### 3.3.3 Midkine and other neuropeptides

Midkine, a heparin-binding growth factor, contributes to a neuron-immune-cancer axis that supports tumor progression. In low-grade gliomas, midkine activates CD8^+^ T cells to produce CCL4. This CCL4 then stimulates microglia to secrete CCL5, a factor crucial for glioma stem cell survival (50). Elevated CCL5 correlates with reduced patient survival, highlighting the clinical relevance of this pathway. This intricate signaling cascade highlights that CD8^+^ T cells are co-opted by midkine to indirectly support tumor growth through a microglia-mediated pathway (50).

Other neuropeptides, such as vasoactive intestinal peptide (VIP) and neuropeptide Y (NPY), also modulate immune responses. VIP is produced primarily by neurons and immune cells and acts on receptors (VPAC1/2) expressed on T cells, macrophages, and DCs, generally promoting anti-inflammatory and tolerogenic states that can facilitate immune evasion (49). NPY, co-released with NE from sympathetic nerve terminals, signals through Y1-R and other receptors on endothelial cells and various immune cells, influencing angiogenesis, immune cell trafficking, and can exert either stimulatory or suppressive effects on immunity depending on the context (49). As highlighted here, the effects of these peptides are highly context-dependent, varying with tumor type, microenvironment, and receptor expression patterns on target cells.

## 4 Neural regulation of specific immune cell populations in the TME

**Figure 3:**
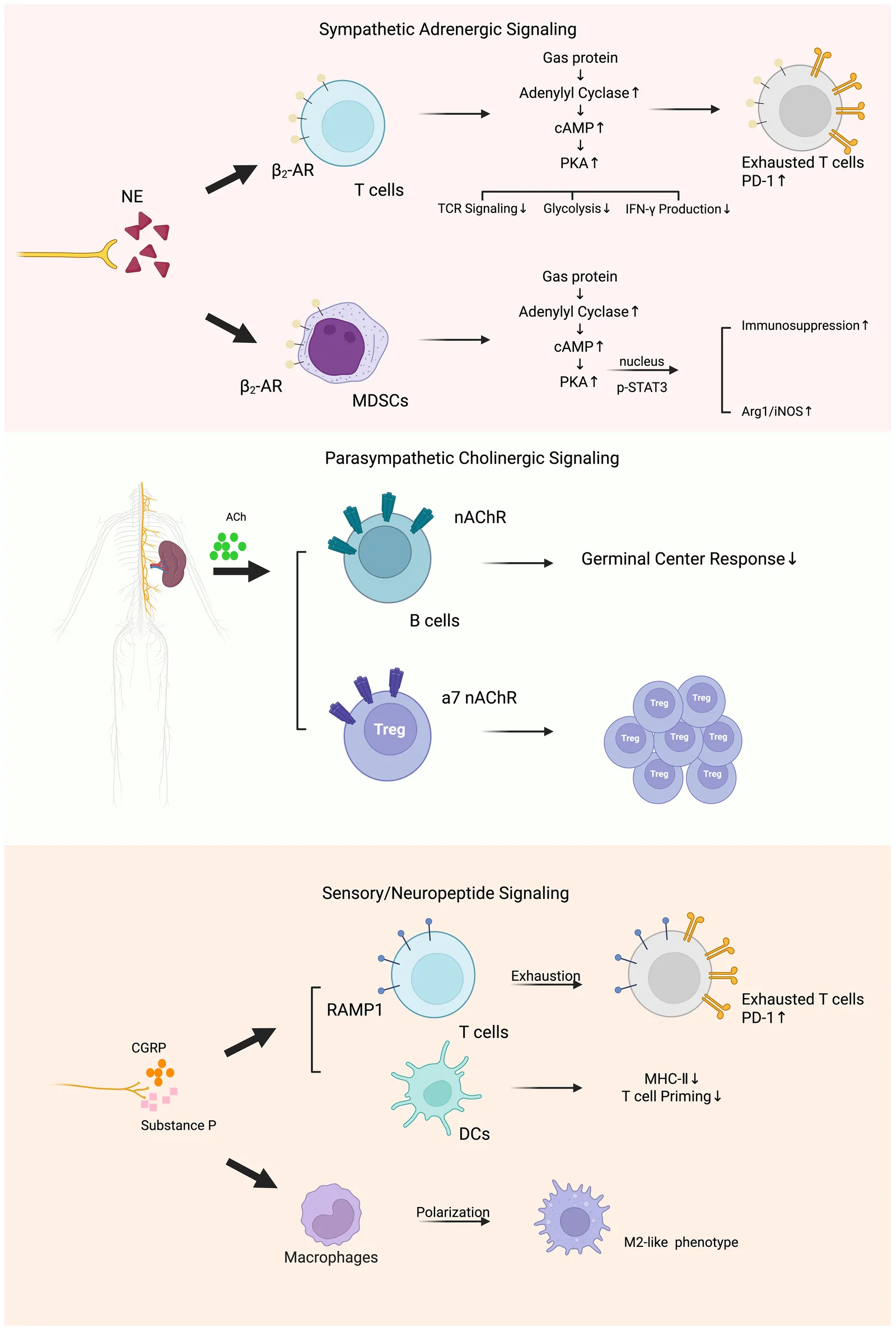
Neural-immune crosstalk in the tumor microenvironment. This schematic summarizes three major neural signaling pathways that suppress anti‑tumor immunity in the TME. 1) Adrenergic Signaling:​ NE released from sympathetic nerve terminals binds to β_2_-AR on immune cells. In CD8^+^ T cells, this activates a Gαs‑cAMP‑PKA cascade, resulting in metabolic dysfunction (reduced glycolysis), functional exhaustion (upregulation of PD‑1, CTLA‑4, LAG‑3), and impaired cytotoxicity (decreased IFN‑γ). Concurrently, NE‑driven β_2_‑AR signaling in MDSCs promotes their expansion and enhances immunosuppressive functions via STAT3 phosphorylation and increased arginase‑1/iNOS expression. 2) Cholinergic signaling: ACh from vagal nerve fibers modulates splenic immune responses, attenuating the germinal center reaction by inhibiting nicotinic ACh receptor (nAchR) signaling of B‑cell and expanding regulatory T cells (Tregs) through α7 nAchR. 3) Sensory neuropeptide signaling: Calcitonin gene‑related peptide (CGRP) and substance P released from sensory nerve endings act on receptors such as RAMP1 (for CGRP) on CD8⁺ T cells and dendritic cells (DCs), directly promoting T‑cell exhaustion and inhibiting DC maturation. Collectively, these pathways establish an integrated neuro‑immunosuppressive axis within the TME.

The intricate neuro-immune crosstalk within the TME profoundly impacts the function of various immune cell populations, often skewing their activity towards immune evasion and tumor promotion. As summarized in **Figure 3**, this crosstalk forms a complex network where different neural components (sympathetic, sensory, parasympathetic) signal through specific neurotransmitters and neuropeptides to regulate distinct immune cell subsets and their functions. This section delves into the specific mechanisms by which neural signals modulate key immune cell types, including T cells, macrophages, MDSCs, DCs, and B cells.

### 4.1 T cells

T cells, particularly cytotoxic CD8^+^ T cells, are central effectors of anti-tumor immunity. However, their function is highly susceptible to suppression by neural signals within the TME.

#### 4.1.1 Sympathetic nervous system activity impairs T cell priming and trafficking

The SNS significantly impairs CD8^+^ T cell responses by targeting both their initial activation and their migration to tumors (36, 42). NE, released by sympathetic nerve endings, acts on ARs expressed on T cells and antigen-presenting cells (APCs) within lymphoid organs. This signaling suppresses the priming of naive CD8^+^ T cells, hindering their differentiation into potent effector cells (42). Moreover, SNS activity regulates the egress of primed T cells from draining lymph nodes, a critical step for their recruitment to the tumor site. Interventions that reduce adrenergic signaling or specifically block β_2_-AR have been shown to enhance antigen-specific anti-tumor immunity by facilitating T cell egress from lymph nodes (37).

#### 4.1.2 Adrenergic stress induces T cell exhaustion and metabolic dysfunction

Chronic adrenergic stress, a common feature in cancer patients, directly promotes T cell exhaustion and disrupts their metabolic fitness within the TME (37, 38). Sustained signaling through β_2_-ARs on T cells suppresses T-cell receptor (TCR) signaling and drives a state of functional exhaustion. This exhausted phenotype is characterized by impaired cytokine production and proliferation, coupled with elevated expression of inhibitory receptors such as PD-1, CTLA-4, and LAG-3 (38, 51). This dysfunction is exacerbated by a concurrent metabolic disruption, as adrenergic signaling suppresses both glycolysis and oxidative phosphorylation in T cells (38). Importantly, the administration of β-AR blockers can reverse T cell exhaustion, restore metabolic capacity, and enhance tumor control (38).

### 4.2 Macrophages and MDSCs

Macrophages and MDSCs are pivotal mediators of immunosuppression in the TME, and their functions are profoundly shaped by neural inputs.

#### 4.2.1 Neural signals promote macrophage polarization

TAMs are highly plastic and can adopt an immunosuppressive, pro-tumorigenic M2-like phenotype. Neural signals within the TME actively promote this polarization (6, 8). Neurotransmitters released by tumor-infiltrating nerves can directly act on macrophages, influencing their gene expression profiles and cytokine production. The interplay between neural components and immune/stromal cells in PNI involves critical nodes like CAFs and TAMs, where shared signaling pathways such as CXCL12/CXCR4 and TGF-β are crucial for promoting a “cold” TME and immunotherapy resistance (34). Metabolic reprogramming can be indirectly affected by neural signals that alter the metabolic demands or nutrient availability in the microenvironment (6, 8). For example, lactate, a key metabolite in the TME, can promote tolerogenic dendritic cell maturation and influence macrophage polarization by neural activity (9, 52). Furthermore, efferocytosis, the process of clearing apoptotic cells, can reprogram macrophages to promote pancreatic cancer liver metastasis, with progranulin expression in macrophages being crucial for this conversion to arginase 1-producing cells, a process that could be influenced by neural signals (53).

#### 4.2.2 β-AR signaling enhances MDSC generation and function

The expansion and immunosuppressive potency of MDSCs are significantly augmented by β_2_-AR signaling (39). NE binding to β_2_-ARs on myeloid precursors and immature MDSCs, promotes their differentiation, proliferation, and survival. This leads to an increased frequency of MDSCs in both tumors and lymphoid organs, and upregulates their immunosuppressive machinery, including arginase-1, iNOS, and PD-L1 (39, 41). The underlying mechanism frequently involves the activation of STAT3 phosphorylation. Consequently, MDSCs become more potent in suppressing cytotoxic T cell responses. The β-AR antagonists like propranolol can reduce MDSC numbers and suppress their activity (39).

#### 4.2.3 Neural innervation regulates myeloid cell expansion

Beyond direct intratumoral effects, neural innervation can systemically control myeloid cell expansion through a reflex arc involving the spleen (54). The spleen is a primary site for the expansion of CD11b^+^Gr-1^+^ MDSCs during cancer. A vagus nerve-mediated circuit counteracts this expansion; neural innervation stimulates vagally modulated memory T cells in the spleen to release Trefoil Factor 2 (TFF2). TFF2 then suppresses MDSC expansion via CXCR4 signaling. Disruption of this arc, either through splenic denervation or genetic deletion of TFF2, leads to uncontrolled MDSC expansion and exacerbates colorectal carcinogenesis. Conversely, enhancing TFF2 signaling can suppress tumor development, revealing a sophisticated neuro-immune loop for systemic cancer control (54).

### 4.3 DCs and B cells

DCs and B cells are crucial components of the adaptive immune system, playing distinct but complementary roles in anti-tumor immunity. Their functions, from antigen presentation to antibody production, are also subject to modulation by neural signals.

#### 4.3.1 Dendritic cell trafficking and function are subject to neural control

The trafficking and functional maturation of DCs, critical for priming naïve T cells, are influenced by neural signals (36, 55). Circadian rhythms, which are under neural control, play a significant role in regulating DC trafficking and anti-tumor immune responses. Rhythmic DC trafficking to the tumor-draining lymph node governs a circadian response of tumor-antigen-specific CD8^+^ T cells, dependent on CD80 expression (56). This suggests that the timing of DC activity, dictated by the circadian clock, can profoundly impact the efficacy of anti-tumor immunity and potentially cancer immunotherapy outcomes (56, 57). Furthermore, neurotransmitters and neuropeptides can directly act on DCs, influencing their maturation, cytokine production, and ability to present antigens. For example, CGRP has been linked to abnormal dendritic cell development, impairing their ability to activate T cells (23). The TME itself, through factors like hypoxia, tumor exosomes, and co-inhibitory molecules, can induce DC dysfunction, promoting immune evasion (55). Neural signals, by influencing these TME characteristics or directly acting on DCs, can thus contribute to this dysfunction, further hindering effective anti-tumor responses.

#### 4.3.2 Vagus nerve stimulation modulates B cell activity and antibody production

The vagus nerve, through its cholinergic signaling, has a direct and significant impact on B cell activity and antibody production in lymphoid organs (48). Chronic vagus nerve stimulation (VNS) has been shown to modulate distinct AChRs on B cells, leading to a limitation of the GC response. In mice, VNS results in lower IgG titers, fewer germinal center B cells, and impaired plasma cell differentiation (48). This suggests that sustained cholinergic input can suppress the humoral immune response. According to the earlier, the effects are mediated by specific nAChR subtypes, while α4 nAChR specifically inhibits CD19-B cell receptor colligation, a crucial step for B cell survival and activation (47, 48). In cancer, this neural modulation of B cell activity could have complex implications for the overall anti-tumor immune response (58). Suppressing antibody production or altering B cell differentiation could potentially hinder the development of protective humoral immunity against tumor antigens, thereby contributing to immune evasion.

### 4.4 Other immune cells

Other immune populations within the TME, such as neutrophils, are also susceptible to neuro-immune modulation. Neutrophils are increasingly recognized for their diverse roles within the TME, where they can exhibit both anti-tumor (N1) or pro-tumor (N2) phenotypes (59). Neurotransmitters and neuropeptides can modulate neutrophil trafficking, degranulation, and cytokine production. Melatonin can polarize neutrophils toward an N1-like, anti-tumor phenotype (60). Similarly, neuropeptides released by tumor-infiltrating nerves might directly act on neutrophils, altering their phenotype towards a pro-tumorigenic N2-like state, which promotes angiogenesis, metastasis, and immunosuppression (59). Understanding how neural signals regulate this functional switch may reveal novel therapeutic targets for reprogramming neutrophil activity in the TME.

## 5 Neural circuits regulate antitumor immunity and therapeutic responses

Complex neural circuits, both peripheral and central, exert a systemic regulatory influence over anti-tumor immunity and can significantly impact therapeutic responses (61, 62). The brain, through its intricate neural networks, communicates with peripheral tumors via the peripheral nervous system, endocrine, and immune systems (61).

A specific brain-tumor neural circuit involving the corticotropin-releasing hormone (CRH) neurons in the central medial amygdala (CeM) has been identified to regulate breast cancer progression in mice (62). Tumor burden triggers anxiety-like behaviors in mice, which activates these CeM ^CRH^ neurons. This activation, in turn, influences sympathetic nerves within the tumors, forming a polysynaptic circuit that promotes tumor growth. Inhibition of this circuit slows tumor growth, while its activation accelerates growth, demonstrating a direct link between central neural activity, peripheral sympathetic tone, and tumor progression (62). Functional and anatomical evidence suggests that the efferent pathway likely involves: CeM ^CRH^ neurons projecting to and exciting catecholaminergic neurons in the LPGi, which in turn innervate sympathoadrenal preganglionic neurons in the spinal cord; this ultimately leads to increased noradrenergic output from the postganglionic sympathetic fibers that infiltrate the tumor stroma (62). Furthermore, manipulating this CeM ^CRH^→(lateral paragigantocellular nucleus) LPGi circuit regulates intratumoral sympathetic nerves and peripheral NE levels, which consequently affects anti-tumor immunity.

Other central circuits also exert antitumor effects. Activation of hypothalamic oxytocin (Oxt) neurons slows colorectal cancer progression by suppressing sympathetic neuronal activity in the celiac-superior mesenteric ganglion (CG-SMG) complex, which reduces peripheral NE levels (63). Similarly, activation of the brain’s reward system reduces tumor burden by dampening sympathetic drive, which diminishes immunosuppressive MDSCs in the bone marrow and boosts systemic immune function (64).

Beyond these connections, the central nervous system also coordinates immune function through circadian rhythms. This approximately 24-hour cycles that regulate many physiological processes, are deeply intertwined with immune function and are under the control of the central nervous system’s master clock (57, 65). Within the TME, these rhythms play a critical role in directing anti-tumor immune responses, particularly through their influence on DCs (56). Research has demonstrated that rhythmic trafficking of DCs to the tumor-draining lymph node governs a circadian response of tumor-antigen-specific CD8^+^ T cells, a process dependent on CD80 expression (56). Disruption of these circadian rhythms, often seen in cancer patients, can therefore lead to a dysregulated immune microenvironment that favors tumor progression (66, 67).

**Figure 4:**
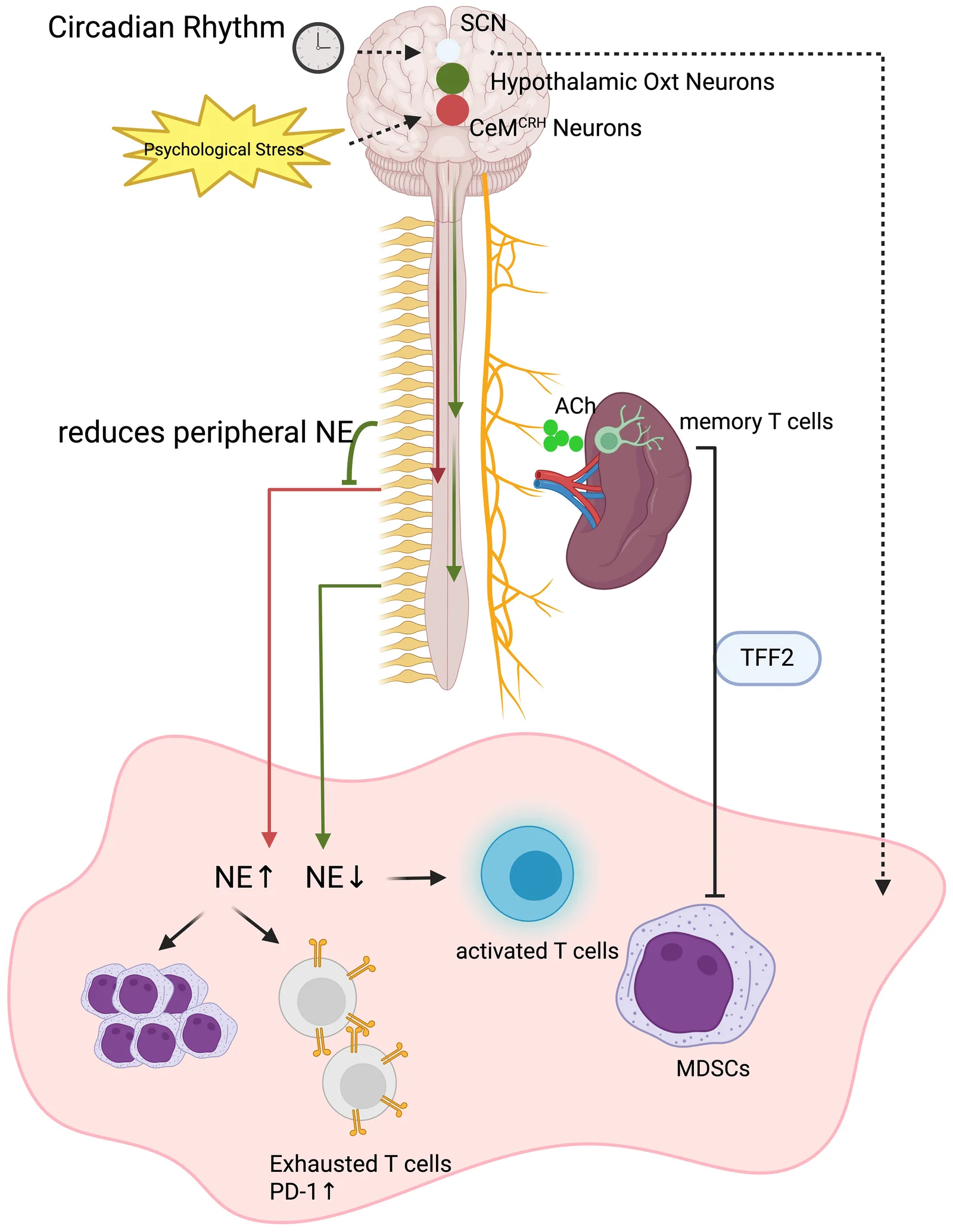
Central neural circuits regulate peripheral antitumor immunity. Central neural circuits regulate peripheral antitumor immunity. This schematic depicts the brain–body axis that modulates tumor progression through neuroimmune pathways. Key central nuclei are identified: the suprachiasmatic nucleus (SCN) controlling circadian rhythms, the Oxt neurons constituting an inhibitory circuit, and the CeM^CRH^ neurons activated by psychological stress​ forming a promoting circuit. The CeM^CRH^ circuit increases sympathetic output, elevating NE levels in the TME, which leads to T cell exhaustion and MDSC accumulation. Conversely, the Oxt neuron circuit reduces peripheral sympathetic activity (reduced NE). Also illustrated is a distinct splenic neuro‑immune reflex, in which vagus nerve‑derived ACh acts on splenic memory T cells to induce release of trefoil factor 2 (TFF2), which in turn can suppress MDSC activity.

These findings reveal that psychological states and central neural circuits can profoundly impact anti-tumor immunity and cancer progression, offering novel avenues for therapeutic intervention that target the brain-body axis to enhance systemic anti-tumor responses (**Figure 4**).

## 6 Therapeutic implications and future directions

The growing understanding of the neuro-immune axis in cancer has opened new therapeutic avenues that extend beyond traditional cancer cell-centric approaches, focusing on the complex interactions between the nervous and immune systems in the TME. These insights offer opportunities to overcome therapeutic resistance and enhance anti-tumor immunity.

### 6.1 Targeting the neuro-immune axis

Recognizing nerves as active participants in cancer progression and immune modulation has led to innovative strategies targeting the neuro-immune axis (13, 14, 20). These strategies aim to disrupt pro-tumorigenic neural signals and reprogram the immune microenvironment to improve patient outcomes.

#### 6.1.1 Denervation and neurotransmitter receptor blockade

Disrupting the nerve supply to tumors, either surgically or pharmacologically, represents a direct intervention. Denervation has been shown to reverse CINI-driven resistance to anti-PD-1 therapy (18). Pharmacological strategies, such as β-AR blockade with propranolol, have demonstrated anti-tumor effects by reducing angiogenesis, enhancing T cell responses, and mitigating MDSC immunosuppression (39, 42, 43). Additionally, targeting neuropeptide receptors, like the CGRP receptor, has shown promise in limiting tumor growth and reducing T cell exhaustion (22, 23).

A major hurdle in repurposing drugs like propranolol is their systemic action. Non-selective β-blockers can cause cardiovascular and metabolic side effects, which may limit their tolerable dosing in cancer patients. Furthermore, the heterogeneity of sympathetic innervation and adrenergic signaling across different cancer types (e.g., prostate versus pancreatic cancer) suggests that patient stratification and the development of more tumor-specific neural blockers are critical for clinical success.

#### 6.1.2 Repurposing neuroactive drugs

Repurposing existing neuroactive drugs, such as beta-blockers, is an attractive strategy due to their established safety and pharmacokinetic profiles (20). Beta-blockers, for instance, improve T cell priming and reduce MDSC immunosuppression, making them ideal candidates for combination therapy with ICIs (42, 43). Other neuroactive compounds targeting neurotransmitter systems, such as dopamine, NE, or serotonin pathways, are being explored for their potential to influence the TME and enhance immune escape (19). Clinical trials are investigating the efficacy of combining these drugs with ICIs to overcome resistance (15, 20).

#### 6.1.3 Modulating central nervous system activity

Modulating CNS activity to combat cancer is a novel therapeutic avenue. For instance, activation of brain circuits, such as the reward system, has been shown to enhance anti-tumor immunity and reduce tumor mass by decreasing immunosuppressive MDSCs (64, 68). Stimulating Oxt neurons can suppress CRC progression by modulating sympathetic neuronal activity (63). In glioblastoma, targeting neuron-glioma interactions, such as thrombospondin-1 with gabapentin, has shown promise in reducing tumor proliferation (69).

### 6.2 Overcoming immunotherapy resistance

Immunotherapy, particularly ICIs, has revolutionized cancer treatment, but resistance remains a challenge (19, 70). The neuro-immune axis plays a significant role in this resistance, providing new strategies to address these barriers.

#### 6.2.1 Neural regulation contributes to resistance to ICIs

Neural regulation fosters an immunosuppressive TME that contributes to ICI resistance. CINI, characterized by nerve myelin degradation and chronic inflammation, impairs anti-PD-1 therapy response (18). Neurotransmitters, such as dopamine, NE, and serotonin, influence the TME, promoting immune escape and contributing to resistance (19). High intratumoral nerve density, associated with PNI, limits immune cell infiltration and function, further enhancing ICI resistance (12, 34).

#### 6.2.2 Combining neuro-modulation with immunotherapy

Given the role of neural pathways in ICI resistance, combining neuro-modulation with immunotherapies presents a promising approach to enhance efficacy (18, 19). For example, denervation or targeting neural pathways like Atf3 or Ifnar1 can reverse CINI-driven resistance to anti-PD-1 therapy (18). Combining anti-PD-1 with anti-IL-6-receptor blockade also improves outcomes (18). Additionally, β-AR blockade with propranolol enhances anti-CTLA4 therapy efficacy by increasing T cell infiltration and reducing MDSCs (43).

### 6.3 Challenges and future research directions

Despite advancements, several challenges remain in translating neuro-immune insights into effective clinical interventions. The complexity of the neuroimmune crosstalk requires advanced technologies like single-cell RNA sequencing (scRNA-seq) and spatial transcriptomics to resolve cell-specific interactions and heterogeneity (71–73). Moreover, developing targeted drug delivery systems responsive to TME characteristics, such as hypoxia, and leveraging the circadian rhythm of anti-tumor immunity for chronotherapy, could optimize therapeutic efficacy (56, 57, 74–76).

Translating preclinical findings into clinical applications remains a major challenge (13, 20). Multi-omics and spatial biology analysis are essential for identifying predictive biomarkers for therapeutic response (71, 77). Clinical trials must address the systemic side effects of neuroimmune interventions, with repurposed neuroactive drugs offering a faster pathway for clinical translation (20).

## 7 Conclusion

The TME is a dynamic ecosystem, with the nervous system playing a critical role in cancer initiation, progression, and therapeutic response. Cancer cells orchestrate tumor innervation through the secretion of neurotrophic factors, exosomes, and CSC-derived neurons, with PNI contributing to aggressive phenotypes and resistance to ICIs by inducing chronic inflammation. Neural signaling, through neurotransmitters like NE and CGRP, suppresses anti-tumor immunity by enhancing MDSC immunosuppression and T cell exhaustion. Conversely, interventions like β-AR blockade enhances T cell responses. Systemic neuro-immune interactions, including brain-body circuits and circadian rhythms, influence tumor progression and immune evasion. Targeting the neuro-immune axis, through denervation, neurotransmitter blockade, or CNS modulation, offers new therapeutic strategies. To translate these insights into clinical breakthroughs, focused efforts are needed in the next five years. Key directions include: applying single-cell and spatial transcriptomics to map the precise cellular interactions at the neuro-immune interface within human tumors; developing innovative preclinical models (e.g., humanized co-culture systems, advanced *in vivo* imaging) to definitively test causal relationships and drug efficacy; designing biomarker-driven clinical trials to identify patients most likely to benefit from neuro-modulatory combination therapies. Above all, neuro-immune axis is as a promising target for improving cancer therapy and overcoming resistance.

## Glossary

TME: tumor microenvironment
MDSCs: myeloid-derived suppressor cells
ECM: extracellular matrix
CAFs: cancer-associated fibroblasts
TAMs: tumor-associated macrophages
PNI: perineural invasion
NGF: nerve growth factor
BDNF: brain-derived neurotrophic factor
GDNF: glial cell line-derived neurotrophic factor
CSCs: cancer stem cells
miRNAs: microRNAs
PDAC: pancreatic ductal adenocarcinoma
EMT: epithelial-mesenchymal transition
CINI: cancer-induced nerve injury
ICIs: immune checkpoint inhibitors
Tregs: regulatory T cells
NE: Norepinephrine
ARs: adrenergic receptors
DCs: dendritic cells
β-AR: beta-adrenergic receptor
iNOS: inducible nitric oxide synthase
ACh: Acetylcholine
nAChRs: nicotinic acetylcholine receptors
mAChRs: muscarinic acetylcholine receptors
IFN-γ: interferon-γ
CGRP: calcitonin gene-related peptide
NK1R: neurokinin-1 receptor
VIP: vasoactive intestinal peptide
NPY: neuropeptide Y
SNS: sympathetic nervous system
APCs: antigen-presenting cells
TCR: T-cell receptor
TFF2: Trefoil Factor 2
VNS: vagus nerve stimulation
CRH: corticotropin-releasing hormone
CeM: central medial amygdala
LPGi: lateral paragigantocellular nucleus
Oxt: Oxytocin
CG-SMG: celiac-superior mesenteric ganglion
CNS: central nervous system.
